# Evaluation of the Digital Ventilated Cage® system for circadian phenotyping

**DOI:** 10.1038/s41598-025-87530-6

**Published:** 2025-01-29

**Authors:** Selma Tir, Russell G. Foster, Stuart N. Peirson

**Affiliations:** Sir Jules Thorn Sleep and Circadian Neuroscience Institute, Kavli Institute for Nanoscience Discovery, Nuffield Department of Clinical Neurosciences, University of Oxford, Dorothy Crowfoot Hodgkin Building, South Parks Road, Oxford, OX1 3QU UK

**Keywords:** Circadian phenotyping, Circadian screen, Circadian disruption, Home cage monitoring, Locomotor activity, Individually ventilated cage, Neuroscience, Circadian rhythms and sleep

## Abstract

The study of circadian rhythms has been critically dependent upon analysing mouse home cage activity, typically employing wheel running activity under different lighting conditions. Here we assess a novel method, the Digital Ventilated Cage (DVC^®^, Tecniplast SpA, Italy), for circadian phenotyping. Based upon capacitive sensors mounted under black individually ventilated cages with inbuilt LED lighting, each cage becomes an independent light-controlled chamber. Home cage activity in C57BL/6J mice was recorded under a range of lighting conditions, along with circadian clock-deficient cryptochrome-deficient mice (*Cry1*^*−/−*^, *Cry2*^*−/−*^ double knockout). C57BL/6J mice exhibited a 24 h period under light/dark conditions, with a free-running period of 23.5 h under constant dark, and period lengthening under constant light. Animals displayed expected phase shifting responses to jet-lag and nocturnal light pulses. Sex differences in circadian parameters and phase shifting responses were also observed. Cryptochrome-deficient mice showed subtle changes in activity under light/dark conditions and were arrhythmic under constant dark, as expected. Our results show the suitability of the DVC system for circadian behavioural screens, accurately detecting circadian period, circadian disruption, phase shifts and mice with clock defects. We provide an evaluation of the strengths and limitations of this method, highlighting how the use of the DVC for studying circadian rhythms depends upon the research requirements of the end user.

**Supplementary Information:**

The online version contains supplementary material available at 10.1038/s41598-025-87530-6.

## Introduction

Circadian rhythms are approximately 24-hour cycles of physiology and behaviour that occur in most living things. These biological oscillations allow organisms to anticipate daily changes in the environment, adjusting physiology to the varied demands of the 24-hour day. Light is the primary time cue for entraining these rhythms in most organisms. In mammals, light is detected by the eyes and transduced into neural signals that synchronize the master circadian pacemaker located in the hypothalamic suprachiasmatic nuclei (SCN). The SCN coordinates peripheral clocks distributed across tissues and organs, thereby optimizing local physiology^1,2^. This response to light is mediated by retinal photoreceptors, including the rods and cones primarily associated with vision, and photosensitive retinal ganglion cells (pRGCs) expressing the blue-light sensitive photopigment melanopsin^3,4^. As such, light detection by the eye plays a dual role, essential not only for vision, but also for the entrainment of the circadian system, as well as other non-visual responses to light^5–7^. Circadian rhythms are crucial for synchronizing most physiology and behaviour, including sleep-wake cycles, hormone secretion, metabolism, and gene expression^8–10^. Under normal conditions, consolidated, robust, and stable rhythms are observed. However, circadian rhythms can become misaligned or disrupted with respect to the external day/night cycle under conditions that include disease and abnormal exposure to light. Such disruption can be characterized by disturbances in the timing, amplitude, or synchronization of these rhythms, and has been identified as a common comorbidity in various health conditions, including mood disorders and cardiovascular diseases^11–14^. Studying the mechanisms underlying circadian rhythms and their disruption is therefore crucial for understanding disease pathophysiology and for the development of targeted therapeutic interventions.

The effects of light on the circadian system are complex, contingent upon multiple factors including the intensity, wavelength, duration, and timing of light exposure. Furthermore, light history and age can modify circadian responses to light^15–17^. As a result, different lighting conditions can be used to identify the distinct components contributing to circadian entrainment. Standard light-dark (LD) cycles are typically used to assess the phase of entrainment, period (*tau*), activity bouts, and the robustness of behavioural rhythms, and provide a baseline for the study of circadian rhythms in response to different environmental conditions. The assessment of circadian rhythmicity can only be determined in the absence of environmental time cues (known as zeitgebers, or “time-givers”). Constant darkness (DD) allows for the measurement of free-running periods and is particularly useful in identifying clock mutants, where the endogenous circadian period may be longer or shorter than normal, or even arrhythmic. Conversely, constant light (LL) leads to period lengthening in a dose dependent manner, and can even induce arrhythmicity^18^. As such, LL conditions provide valuable insights into the sensitivity of the central pacemaker to light of different intensities and wavelengths^19^. A light pulse (LP) administered during the subjective night can shift the circadian clock, with the magnitude and direction of the subsequent phase shift dictated by the intensity and timing of the light exposure. A LP administered at the beginning of the night will induce a phase delay, while a LP at the end of the night will prompt a phase advance of activity onsets^20^. Phase shifting responses to light played an important role in the identification of the melanopsin-based pRGCs, particularly via action spectroscopy methods employing different wavelengths and intensities of light^21–23^. Another approach to studying phase shifting and responses to circadian challenges, is the use of abrupt shifts of the LD cycle, or “jet lag” protocols. In addition, an animal may appear to entrain to a LD cycle, while activity may simply be suppressed during the light phase of the LD cycle (negative masking)^24,25^. For example, *Cry1*^*−/−*^*/Cry2*^*−/−*^ double knockout mice appear to display normal activity under LD conditions, but become arrhythmic under DD, as they lack a functional clock^26^. Understanding circadian rhythms in mice therefore requires the study of behaviour under a range of lighting conditions, to determine how animals respond under entrained, free-running and shifted LD cycles.

Screening circadian behaviour is not straightforward and requires a comprehensive assessment of an animal’s locomotor activity as a behavioural output of the underlying clock mechanisms. This approach requires a detailed phenotyping screen to define any underlying disruptions to the clock^27–29^. Typically, this involves the study of home cage activity under specific lighting conditions, including LD cycles, DD, and LL, as well as assessments of phase shifting responses^30^. Standard metrics are then computed to determine circadian period (*tau*), as well as measurements of circadian rhythm disruption, including measures of the fragmentation, strength, regularity, and day-to-day stability of the rhythms under different lighting conditions^14^. Wheel-running behaviour has historically been the most common methodology for assessing circadian rhythms^27,31–34^. The pioneering studies of Curt Richter showed that rodents have a natural tendency to engage with running wheels, making it a convenient and efficient method for monitoring voluntary activity patterns over extended periods^35^. This method has a high signal to noise ratio, resulting from minimal activity during the rest phase. This facilitates the identification of activity onsets and circadian phase under LD, DD, and LL, or following a brief light exposure in DD^28^. Wheel-running behaviour has been showed to be effective in identifying phenotypic differences based on mouse strains and age^36–38^, as well as playing a critical role in the identification of mammalian clock mutants^39^. However, studies have shown that the introduction of running wheels can alter behavioural responses in rodents, including changes in food intake^40^, depression-like behaviour^41^, and aggression^42^. In addition, voluntary running wheel activity can affect other aspects of circadian or sleep physiology, notably strengthening circadian rhythms in aging mice^43^, improving circadian disruption^44^, and affecting sleep^45–47^. In addition, wheel-running activity does not capture other behaviours such as grooming, feeding, anticipatory behaviour preceding the dark phase, or other activity during the light phase. Finally, sleep/wake behaviour cannot be inferred using wheel-running. By contrast, mouse immobility has been correlated with sleep bouts using video tracking and passive infra-red (PIR) based systems^48–50^.

In recent years, home cage monitoring techniques have undergone substantial improvements, driven primarily by welfare and behavioural research initiatives, facilitating continuous and non-invasive monitoring of animal behaviour, and often accompanied by real-time data analysis. In addition to running wheels, these systems include PIR sensors, video tracking, radiofrequency identification (RFID) systems, and capacitive sensors^51^. As a result, researchers can choose the most appropriate monitoring method based upon their specific research questions, experimental design, and laboratory constraints. Home cage activity monitoring presents several challenges and limitations. Technical issues such as sensor malfunction, data loss, and environmental disturbances can affect the reliability and reproducibility of the results. By leveraging capacitive sensor technology and real-time data analytics, the Digital Ventilated Cage (DVC) system (Tecniplast SpA, Italy), presents a novel solution to these challenges. The DVC system is based upon widely used individually ventilated cages (IVCs) placed in a rack that continuously records home cage activity^52^. It comprises capacitive sensor boards equipped with 12 equally spaced electrodes, which detect the presence of animals based on changes in capacitance across electrodes. This provides an animal locomotion index measuring cage level activity. Clear and red IVCs can be used to entrain animals to the room light cycle, while black IVCs equipped with lighting systems (Leddy) can provide individual LD cycles in every cage. As well as activity inside the cage, the DVC system can record when cages are removed from the rack as well as information regarding environmental room conditions outside the cage, enabling potential sources of disturbance to be monitored. Data are available and stored in real-time on the cloud-based DVC Analytics platform, which also provides visualization and data analysis algorithms. The DVC system has been used across various fields of mouse biomedical research. The animal locomotion index was used to identify bouts of rest and physical activity in C57BL/6J mice^53^, to analyse outcomes in a stroke mouse model^54^, and to characterize rest and activity phenotypes in a mouse model of amyotrophic lateral sclerosis (ALS)^55^ and myotonic dystrophy type 1^56^. The DVC system has also been employed to investigate the effects of cage change (introduction of fresh bedding)^52,57^ and the position of a cage in a rack on activity rhythms^58^, as well as the associations between activity and recovery from an Achilles tendon injury^59^. The spatial resolution of the DVC further allowed differences in home-cage behaviour preferences to be determined between three mouse strains^60^. However, despite its increasing use, to date, no studies have investigated whether the DVC system is suitable for conducting detailed circadian experiments.

Here we present new data exploring the use of the DVC system for circadian phenotyping. Mouse home cage activity was recorded under 12:12 LD, DD, and LL, as well as a 6-hour phase advance, and DD following exposure to a LP to measure phase shifting responses. Key circadian parameters and measures of circadian disruption were then determined under each lighting condition. To define the effectiveness of the DVC system in detecting phenotypic differences, mice lacking a functional circadian clock (*Cry1*^*−/−*^, *Cry2*^*−/−*^) were investigated under 12:12 LD and DD conditions. The DVC provides a novel approach to studying circadian rhythms in mice under IVC husbandry conditions, and combined with black cages and LED lighting can replicate the wide range of lighting conditions used in standard circadian phenotyping screens.

## Methods

### Animals

All animals were single housed under a 12:12 LD cycle for a minimum of one week prior to the start of experiments. *Ad libitum* access to food (Envigo 2916) and water was provided throughout. Wild-type C57BL/6J animals were purchased from Envigo, UK, while Cryptochrome 1 and 2 double knock-out (*Cry1*^*−/−*^, *Cry2*^*−/−*^) animals^26^ were bred in-house on a C57BL/6J background. This study is reported in accordance with the ARRIVE guidelines. All animals were included and were between 3 and 4 months old at the beginning of the study. Control and experimental groups were subjected to the same conditions at the same time to reduce confounding variables. As such, no randomisation or blinding was used. All experimental procedures were carried out at the University of Oxford, UK, in accordance with the University of Oxford Policy on the Use of Animals in Scientific Research and the United Kingdom Animals (Scientific Procedures) Act 1986, under Project License PP0911346 and Personal License I34186130.

### Lighting and housing conditions

**Fig. 1:**
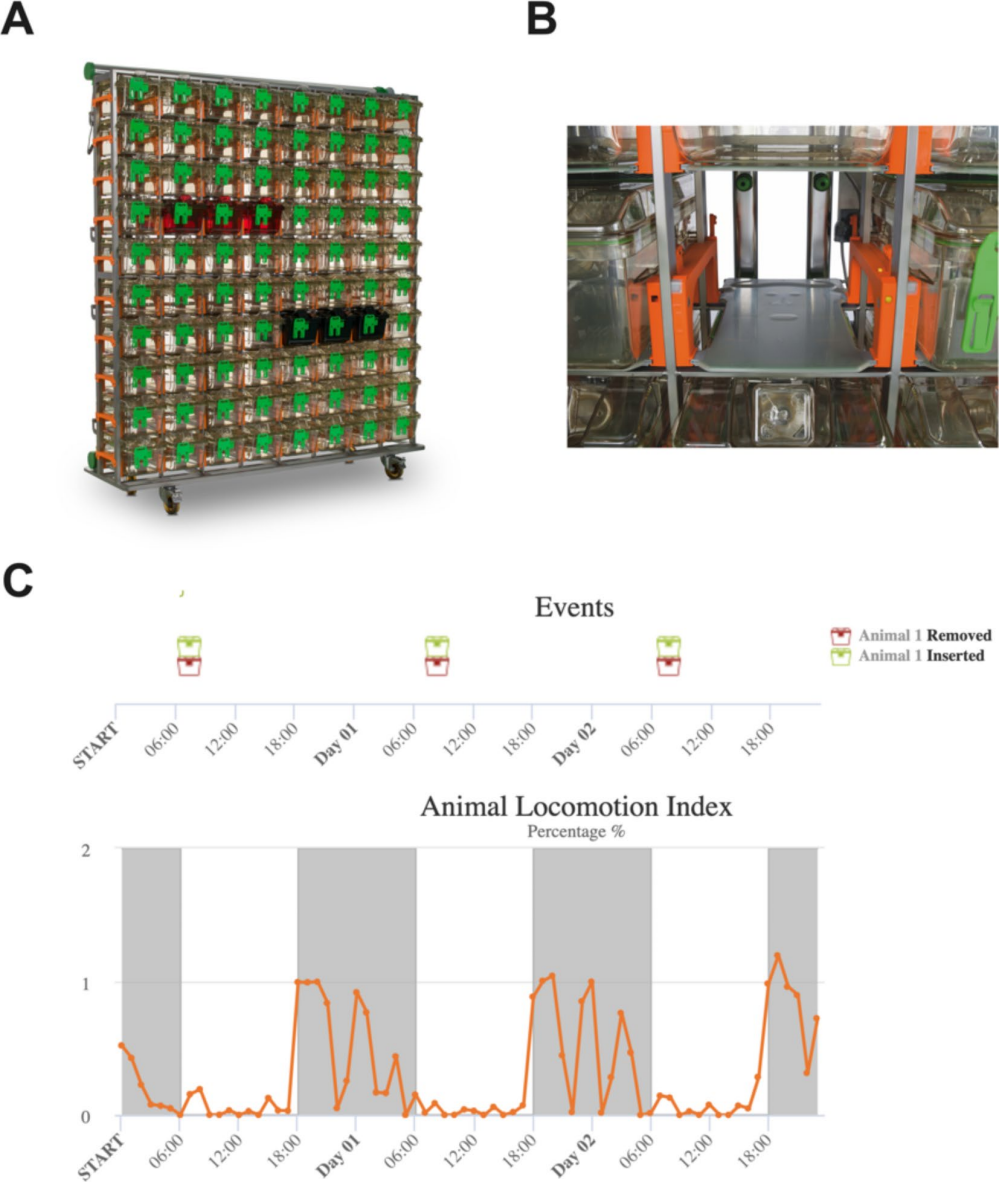
The DVC system and the Animal Locomotion Index. (**A**) DVC rack equipped with clear, red, and black cages. (**B**) Capacitive sensor board. (**C**) Animal locomotion index for one wildtype C57BL/6J mouse, binned by hour, visualized on the cloud-based DVC Analytics platform. Timings of cage insertion and extraction for daily checks are also shown. Dark shading indicates dark phases.

The DVC system (Tecniplast SpA, Italy) allows for the continuous recording of home cage locomotor activity^52^. The system comprises 60 or 80 individually ventilated cages (IVCs) of type GM500, made of clear, red or black polycarbonate, offering options for both single and group housing (Fig. 1A). In this study, we utilised black IVCs, which were equipped with individual cool white LED systems known as Leddy. The Leddy lighting system facilitated the entrainment of animals to specific lighting cycles within each cage, eliminating the need for light-tight chambers typically used in circadian and sleep research. Light levels were kept at ~ 100 photopic lux at the centre of the cage (Leddy light intensity level 22), giving < 2 S-cone opic lux, 70 melanopic lux, 75 rhodopic lux and 78 M-cone opic lux^61^ (Supp. Fig. 1; Supp. Table 1).

### Home-cage locomotor activity monitoring

Each IVC was positioned on a capacitive sensor board featuring 12 evenly spaced electrodes (Fig. 1B). This sensor board detected the presence of an animal by monitoring changes in capacitance across electrodes, primarily due to the animal’s physiological composition (70% water) and movement. Subsequently, an Animal Locomotion Index (ALI) was calculated. This is based on the number of electrodes activated, with 0% = no electrodes and 100% = all 12 electrodes simultaneously activated within the time bin (Fig. 1C). The DVC Analytics platform provided real-time access to the data for visual inspection and download. The system’s default minimum activity binning was set to 1 min, although a resolution of 250 msec is possible. We performed daily welfare checks, with the cages being taken out of the rack for animal inspection for less than 5 min.

### Circadian phenotyping

A total of 12 WT mice (C57BL/6J, 6 males, 6 females) were included in the circadian screen. Home cage locomotor activity was recorded continuously for 10 weeks under various lighting conditions, including 12:12 LD, DD, LL, LD following a 6-hour phase advance (jet-lag), and DD after exposure to a light pulse (LP) at ZT14-16 to measure phase shifting responses. A cage change was performed after the first period of DD (day 29), and before animals were re-entrained to a 12:12 LD cycle.

### Clock-deficient mice

*Cry1*^*−/−*^,*Cry2*^*−/−*^ double knockout mice (*N* = 6, 3 males, 3 females, *N* = 6 C57BL/6J controls) animals were used as a circadian clock deficient model. Cryptochrome-deficient mice are characterized by the deletion of the core clock genes Cryptochrome 1 and 2^26^. These mice have no circadian clock and exhibit arrhythmic behaviour under constant conditions. This animal model was employed to assess whether the DVC system could effectively detect phenotypic differences based on locomotor activity, particularly under LD and DD conditions (1 week each).

### Statistical analyses

Raw animal locomotion index data were exported from the DVC Analytics platform in 1 min and 1 h bins. Chi-square periodogram and activity onsets were computed in ActogramJ^62^. A 30-min running average was applied in order to account for missing values (daily checks) and to compute actograms. Inter-daily stability, intra-daily variability and relative amplitude were coded in R based on the equations provided in Witting et al.^63^. Light phase activity and total activity were calculated in R. One week of data was used to compute the circadian disruption measures for each lighting condition. Due to a user error, one female mice is missing the final 17 h of activity recording during the DD period. Statistical testing and plotting were conducted in Prism 10. One-way or two-way repeated-measures ANOVA, with a Geisser-Greenhouse correction (lack of sphericity) and Holm- Šídák’s multiple comparisons test were computed. When comparing two groups, unpaired nonparametric Mann-Whitney tests or Wilcoxon matched-pairs signed rank tests were used, as the distribution of the data was not Gaussian. α = 0.05 was adopted in all analyses. Mean ± standard error of the mean (SEM) was used for all plots.

## Results

### Circadian screen

**Fig. 2:**
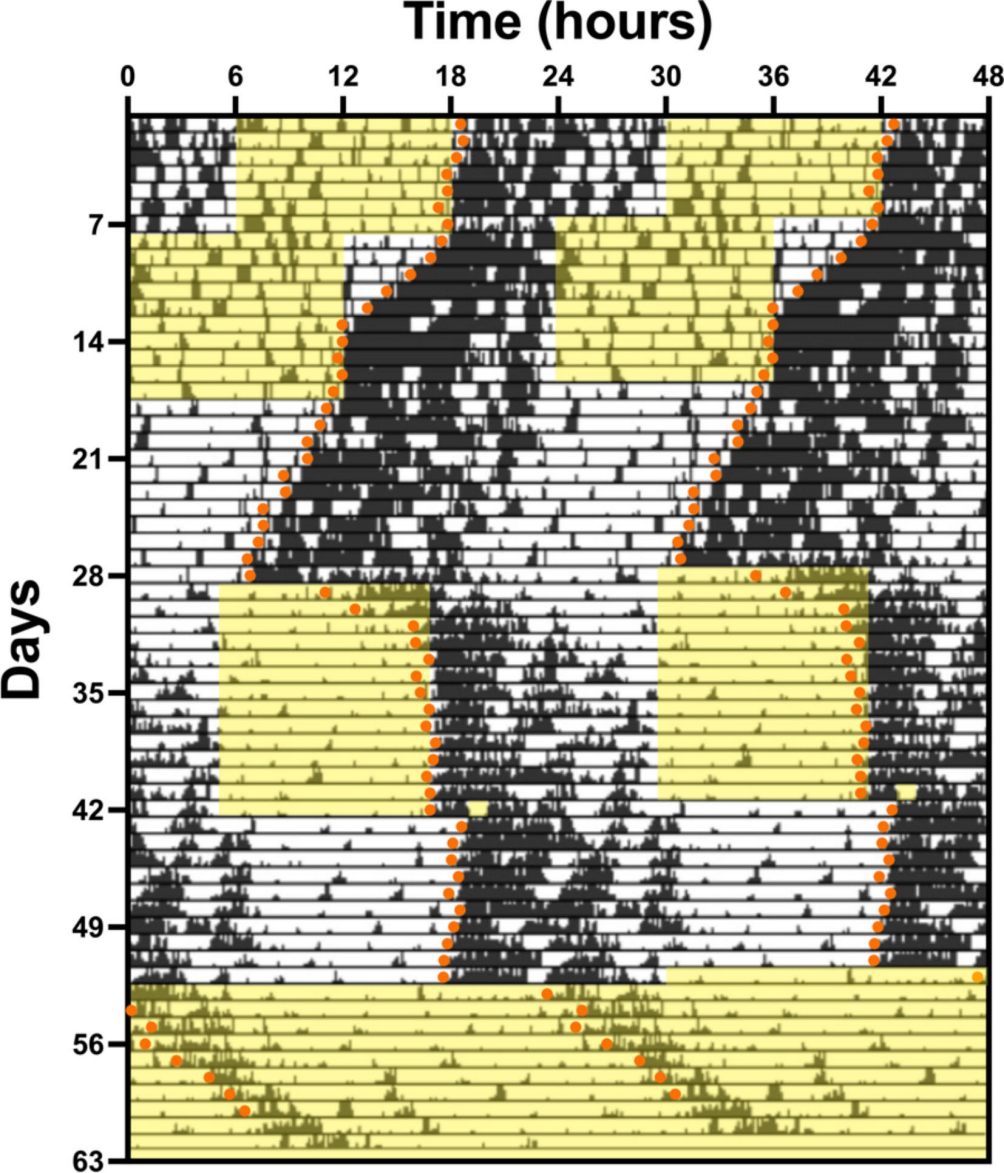
Measuring circadian rhythms with the DVC system. Representative double-plotted actogram of locomotor activity for a wildtype C57BL/6J mouse using the DVC system. The circadian screen included 1 week in 12:12 Light-Dark (LD), 10 days in LD following a 6-hour advance, 10 days in constant darkness (DD), 2 weeks in LD to re-entrainment, 10 days in DD following a LP at ZT14-16, and 10 days in constant light (LL). Yellow shading indicates light phases, while orange dots depict activity onsets.

To assess the suitability of the DVC system for circadian phenotyping studies, mice were singly housed in DVCs, and locomotor activity was monitored under LD, DD, LL, a 6-hour phase advance, and DD following a LP. A representative double plotted actogram is shown in Fig. 2 and illustrates entrainment to the various light-dark cycles, with orange dots indicating activity onsets. Home cage locomotor activity rhythms in LD, DD and LL, were further analysed using standard measures of circadian disruption^14^.

### Periodogram analysis

**Fig. 3:**
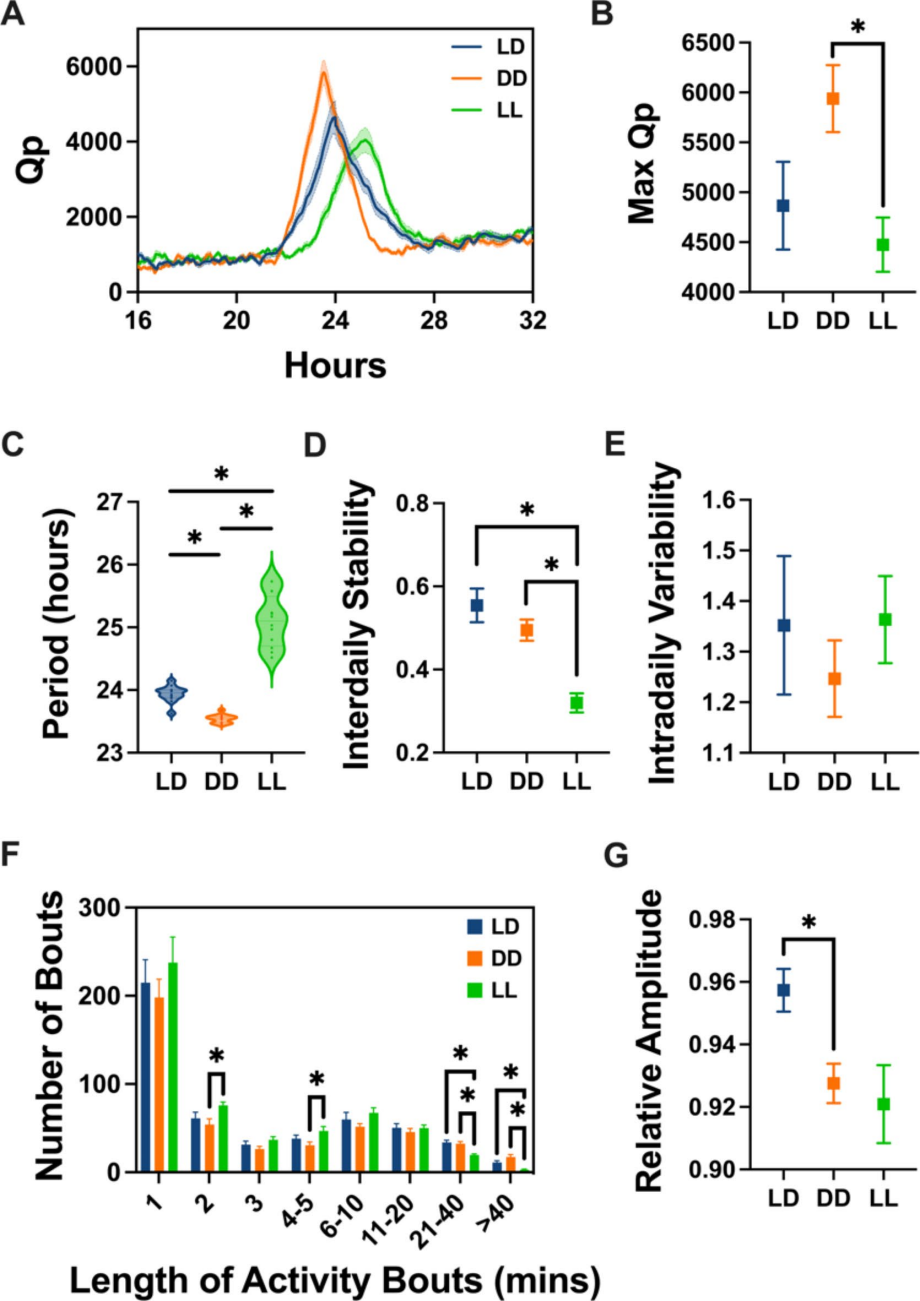
Measures of circadian disruption for 12 wildtype C57BL/6J mice under 12:12 Light-Dark (LD), constant darkness (DD) and constant light (LL). (**A**) Chi-square periodogram analysis. (**B**) Maximum Qp values (Lighting, *F*_(1.565, 17.22)_ = 4.507, *P* = 0.0340). (**C**) Distribution of the number and duration of activity bouts (Lighting x Bout length, *F*_(14, 231)_ = 0.8822, *P* = 0.5789). (**D**) Period of activity rhythms (Lighting, *F*_(1.121, 12.34)_ = 106.3, *P* < 0.0001). (**E**) Inter-daily Stability (Lighting, *F*_(1.437, 15.81)_ = 16.71, *P* = 0.0003). (**F**) Intra-daily Variability (Lighting, *F*_(1.598, 17.58)_ = 0.7966, *P* = 0.4404). (**G**) Relative Amplitude (Lighting, *F*_(1.465, 16.12)_ = 4.942, *P* = 0.0295). Mean +/- SEM. Statistically significant multiple comparisons are indicated by an asterisk.

The Chi-square periodogram was employed to quantify the strength and regularity of the activity rhythms (Fig. 3A), expressed through the Qp value, a ratio of variances^64^. A significant effect of lighting condition on the periodogram power was observed (Lighting, *F*_(1.565, 17.22)_ = 4.507, *P* = 0.0340), with a higher Max Qp value under DD compared to LL (mean diff = 1464, *P* = 0.0047; Fig. 3B), indicative of more robust rhythms in DD. Activity patterns under entrained conditions are typically influenced by environmental cues. Under a 24-hour LD cycle, C57BL/6J mice generally display a 24-hour period. However, in DD, the endogenous circadian period is expressed, resulting in a free-running period of approximately 23.5 h^65^. By contrast, constant light suppresses night-time activity, causing the period of activity to lengthen to more than 24 h^66^. Our results showed that the period statistically differed between lighting conditions (Lighting, *F*_(1.121, 12.34)_ = 106.3, *P* < 0.0001), and aligned with the outlined patterns, revealing mean periods of 23.9 h (SEM = 0.04) under LD, 23.5 h (SEM = 0.02) under DD, and 25.1 h (SEM = 0.12) under LL, with higher variability under LL (Fig. 3C).

### Inter-daily stability

The strength of a rhythm is often associated with its day-to-day consistency and can be measured by its inter-daily stability (IS). IS reveals whether the 24-hour activity pattern remains reproducible from one day to another^63^. Under a 12:12 LD cycle, high IS values are expected due to the presence of consistent time cues. Under DD, and in the absence of such cues, IS is expected to decrease slightly. Under LL, constant light weakens the circadian clock in the SCN, leading to reduced day-to-day reproducibility. Here, IS values decreased accordingly from LD, to DD and LL (Lighting, *F*_(1.437, 15.81)_ = 16.71, *P* = 0.0003; Fig. 3D).

### Intra-daily variability

Intra-daily variability (IV) quantifies the fragmentation of activity rhythms, reflecting the number of transitions between activity and rest throughout the day^63^. Our data showed that activity rhythms remained consolidated, with no significant differences between lighting conditions (Lighting, *F*_(1.598, 17.58)_ = 0.7966, *P* = 0.4404; Fig. 3E).

### Activity bouts

The overall number and distribution of activity bouts did not statistically differ between lighting conditions (Lighting x Bout length, *F*_(14, 231)_ = 0.8822, *P* = 0.5789; Fig. 3F). This suggests that the structure of activity patterns was consistent across conditions.

### Relative amplitude

Relative amplitude (RA) is determined by the difference between the period of maximum and minimum activity levels over the 24-hour cycle. It serves as a simple measure of the contrast between periods of high activity and rest^63^. In the absence of external cues, such as DD, the strength of activity rhythms often declines, leading to a decrease in relative amplitude. This decrease tends to be even more pronounced under LL, where light during the subjective night suppresses mouse activity. Consistent with these patterns, here, the relative amplitude varied between lighting conditions (Lighting, *F*_(1.465, 16.12)_ = 4.942, *P* = 0.0295), with a decrease in both DD and LL conditions when compared to LD (Fig. 3G).

### Jet lag

**Fig. 4:**
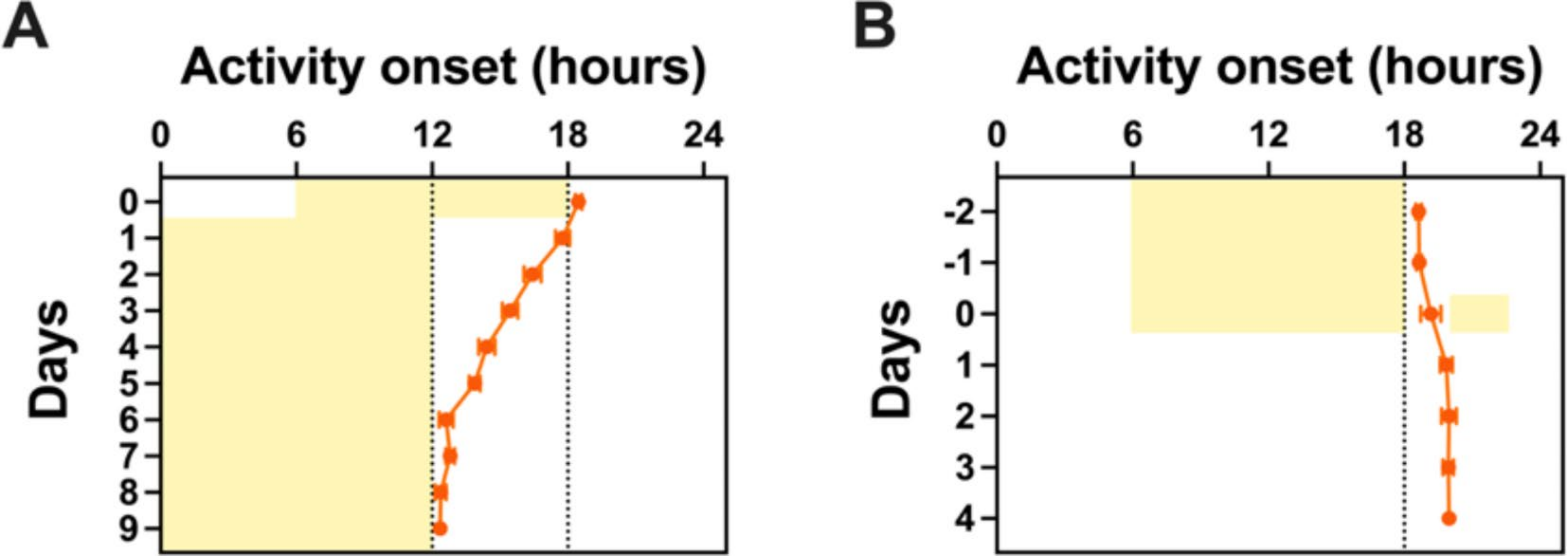
Phase shifting responses for 12 wildtype C57BL/6J mice. (**A**) Animals required about 6 days to re-entrain to the Light-Dark (LD) cycle following a 6-hour phase advance, as illustrated by the shift in activity onset. (**B**) Animals’ activity onsets were delayed by 1.3 h in constant darkness following exposure to a light pulse at ZT14-16 (Wilcoxon matched-pairs signed rank test, Day -1 vs. Day 4, *z* = -1.375, *P* = 0.0005). Mean +/- SEM.

We subjected animals to a 6-hour phase advance of the LD cycle and recorded the duration required for them to re-entrain to the new LD cycle. Results showed that it required 6 days for animals to successfully re-entrain to the LD cycle, suggesting that one day of adjustment is required per hour of phase shift (Fig. 4A).

### Phase shift

Additionally, we investigated whether a LP administered at the beginning of the night (ZT14-16) could shift the phase of activity^28^. When comparing the activity onset of animals one day before the LP (Day − 1) and four days after (Day 4), we observed a delay of about 1.3 h, confirming a shift in the phase of activity consistent with an Aschoff type II protocol (Wilcoxon matched-pairs signed rank test, *z* = -1.375, *P* = 0.0005; Fig. 4B).

### Sex differences

Growing evidence shows that male and female mice may exhibit differences in circadian rhythms. These differences can manifest in various aspects, including the timing and amplitude of activity patterns, sleep-wake cycles, and responses to environmental cues^67^. Despite the tendency of some studies to exclude female cohorts due to concerns about increased variability associated with hormonal fluctuations during estrus, investigating both sexes is essential for gaining a comprehensive understanding of circadian regulation and how it may differ between males and females^68,69^. Subtle differences between the activity profiles of male and female mice were observed (Supp. Fig. 2). We observed a significant main effect of Sex on IV and total activity (IV, *F*_(1, 10)_ = 14.52, *P* = 0.0034; total activity, *F*_(1, 10)_ = 14.85, *P* = 0.0032). Overall, females demonstrated more consolidated rhythms and greater activity levels compared to males. Furthermore, a significant Lighting x Sex interaction was found for both period and RA (Period, *F*_(2, 20)_ = 3.982, *P* = 0.0350; RA, *F*_(2, 20)_ = 4.953, *P* = 0.0179). Females displayed a shorter period (Mean Diff = -0.095) and higher amplitude (Mean Diff = 0.018) under LD, a longer period (Mean Diff = 0.055) and higher amplitude (Mean Diff = 0.006) under DD, and a longer period (Mean Diff = 0.445) and lower amplitude (Mean Diff = -0.045) under LL. The number and distribution of activity bouts differed between sexes under LD (Bout length x Sex, *F*_(7, 70)_ = 4.091, *P* = 0.0008), and LL (Bout length x Sex, *F*_(7, 70)_ = 5.040, *P* = 0.0001), but not under DD (Bout length x Sex, *F*_(7, 70)_ = 1.451, *P* = 0.1991). Under LD and LL, females showed more long bouts of activity. Females also exhibited a greater delay in activity onset in the days following a LP (Sex, *F*_(1, 10)_ = 15.47, *P* = 0.0028), but did not show significant differences in re-entrainment following a 6-hour jet lag (Sex, *F*_(1, 10)_ = 0.5986, *P* = 0.4570; Supp. Fig. 3). Together, these findings highlight the importance of considering both male and female animals in circadian phenotyping studies.

### Clock-deficient mice

In the mammalian circadian clock, CRY1 and CRY2 proteins inhibit the activation of the CLOCK-BMAL1 transcription complex, thereby regulating the expression of clock-controlled genes and ultimately controlling the timing of physiological processes and behaviours^70^. Deletion of both *Cry1* and *Cry2* results in arrhythmic behaviour under constant conditions such as DD^26^. Cryptochrome-deficient mice have also been characterized by abnormalities in cognition and habituation behaviour, and increases in immobility, restlessness and anxiety-like behaviour^71,72^. Here, we investigate whether home cage activity monitoring using the DVC system could detect arrhythmic mouse models.

**Fig. 5:**
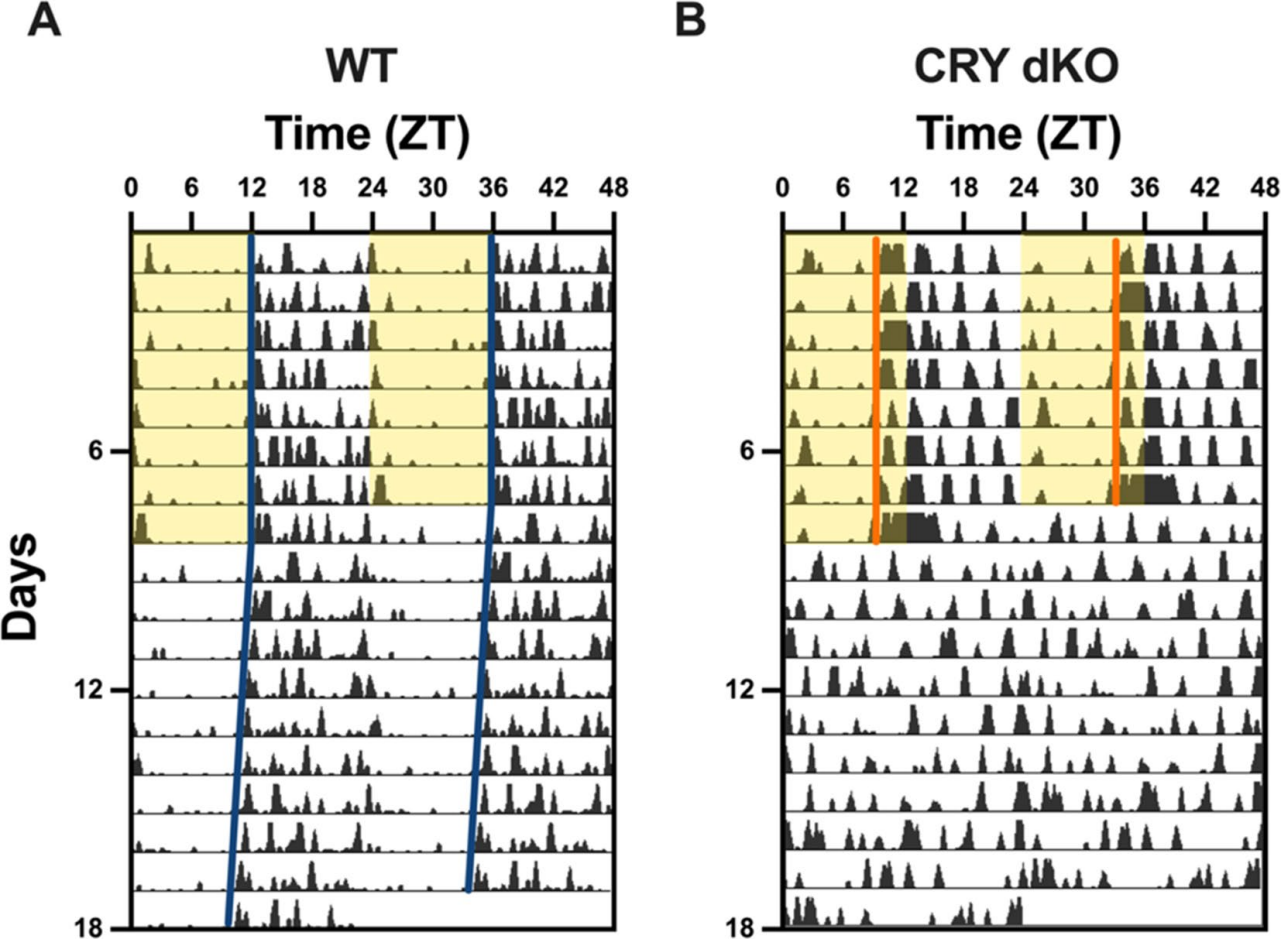
Representative double-plotted actogram of locomotor activity for a **(A)** wildtype C57BL/6J (WT) and a **(B)** Cryptochrome-deficient (*Cry1*^*−/−*^, *Cry2*^*−/−*^, labelled CRY dKO) mouse using the DVC system. Animals were exposed to one week of 12:12 Light-Dark (LD) cycle and 10 days of constant darkness (DD). Yellow shading indicates light phases, while blue and orange lines depict activity onsets.

**Fig. 6:**
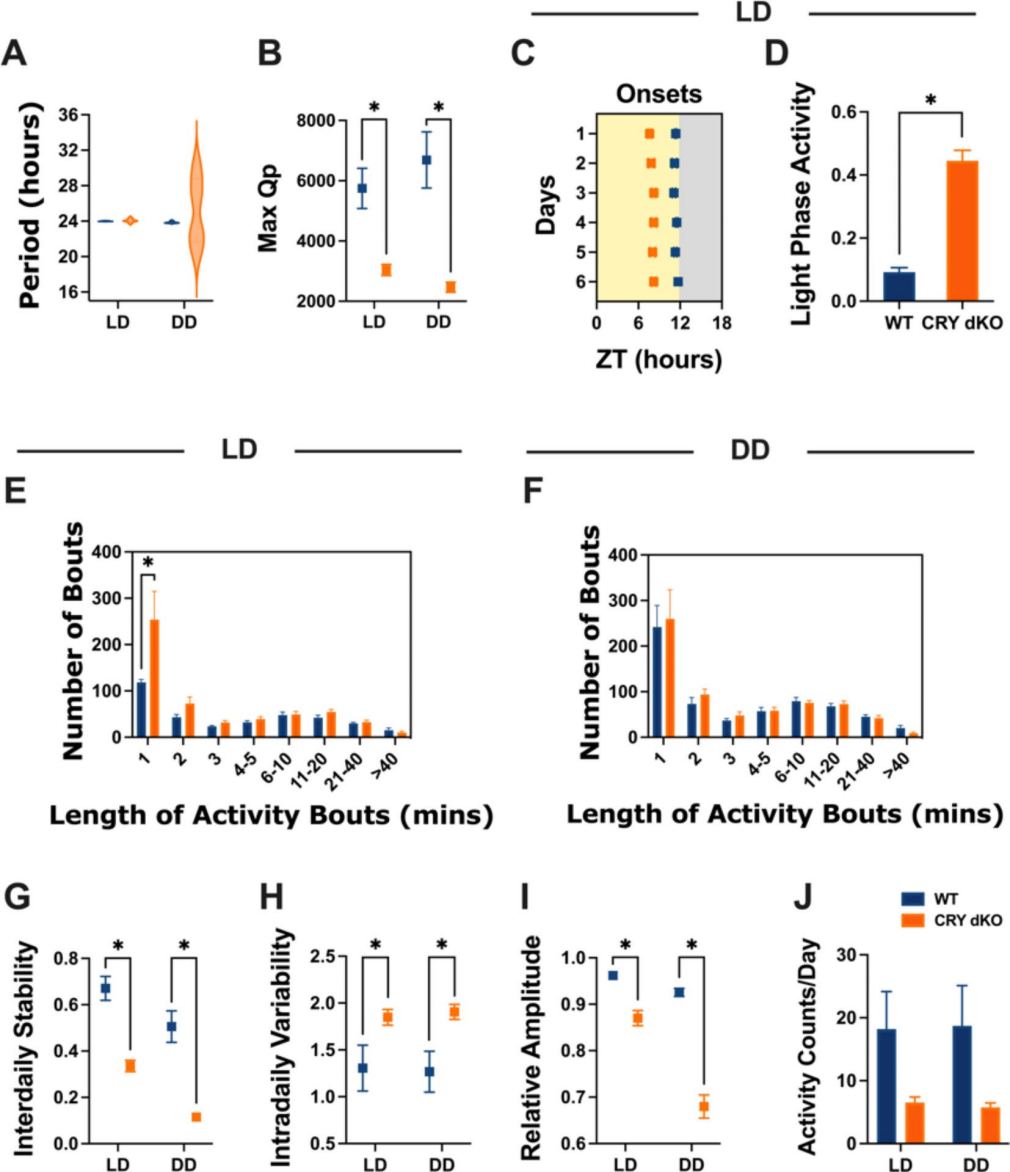
Measures of circadian disruption for 6 wildtype C57BL/6J (WT) and 6 Cryptochrome-deficient (CRY dKO) mice under 12:12 Light-Dark (LD) and constant darkness (DD). (**A**) Period of activity rhythms (Genotype, *F*_(1, 10)_ = 0.6558, *P* = 0.4369). (**B**) Maximum Qp values (Genotype, *F*_(1, 10)_ = 19.31, *P* = 0.0013). (**C**) Activity onsets in LD. (**D**) Proportion of light phase activity in LD. (**E**) Distribution of the number and duration of activity bouts in LD (Bout length x Genotype, *F*_(7, 70)_ = 4.211, *P* = 0.0006). (**F**) Distribution of the number and duration of activity bouts in DD (Bout length x Genotype, *F*_(7, 70)_ = 0.1472, *P* = 0.9938). (**G**) Inter-daily Stability (Genotype, *F*_(1, 10)_ = 38.95, *P* < 0.0001). (**H**) Intra-daily variability (Genotype, *F*_(1, 10)_ = 6.571, *P* = 0.0282). (**I**) Relative Amplitude (Genotype, *F*_(1, 10)_ = 122.6, *P* < 0.0001). (**J**) Total activity per day (Genotype, *F*_(1, 10)_ = 3.969, *P* = 0.0743). Mean +/- SEM. Statistically significant multiple comparisons are indicated by an asterisk.

Representative actograms of cryptochrome-deficient mice show apparently normal nocturnal activity under LD conditions, but arrhythmic activity under DD in comparison with free-running activity in C57BL/6J wildtype mice (Fig. 5). Chi-square periodogram analysis indicated no significant difference in the period of activity rhythms between wildtype and Cryptochrome-deficient animals under 12:12 LD cycles (M = 23.99, SEM = 0.009, and M = 24.06, SEM = 0.078, respectively; Fig. 6A). However, Cryptochrome-deficient animals displayed lower Max Qp values (Genotype, *F*_(1, 10)_ = 19.31, *P* = 0.0013; Fig. 6B), earlier activity onsets (Genotype, *F*_(1, 10)_ = 259.4, *P* < 0.0001; Fig. 6C), more light-phase activity (Mann-Whitney, *U* = 0, *P* = 0.0022, Fig. 6D), and a higher frequency of short bouts of activity compared to wildtype mice (Genotype x Bout length, *F*_(7, 70)_ = 4.211, *P* = 0.0006; 1 min bout, mean diff = -135.3, *P* < 0.0001; Fig. 6E). This suggests that Cryptochrome-deficient animals exhibited less robust and more fragmented rhythms under LD, with increased daytime activity. Under DD, Cryptochrome-deficient mice showed a lack of rhythmicity, as anticipated for animals lacking a functional endogenous clock in the absence of zeitgebers (Fig. 5). Periodogram analysis identified a high variance in their circadian period and lower Max Qp values (Genotype, *F*_(1, 10)_ = 19.31, *P* = 0.0013; Fig. 6B). However, there was no statistical difference between the distribution of their activity bouts compared to WT animals (Genotype x Bout length, *F*_(7, 70)_ = 0.1472, *P* = 0.9938; Fig. 6F). Furthermore, standard measures of circadian disruption highlighted lower IS (Genotype, *F*_(1, 10)_ = 38.95, *P* < 0.0001; Fig. 6G), higher IV (Genotype, *F*_(1, 10)_ = 6.571, *P* = 0.0282; Fig. 6H), and lower RA values (Genotype, *F*_(1, 10)_ = 122.6, *P* < 0.0001; Fig. 6I) in Cryptochrome-deficient mice compared to wildtypes under both LD and DD conditions. These findings suggest less reproducible day-to-day rhythms, increased fragmentation, and weaker amplitude in animals lacking a circadian clock. Although Cryptochrome-deficient animals appeared less active overall, the statistical tests did not reach significance, which is likely due to the wildtype’s high variance (Genotype, *F*_(1, 10)_ = 3.969, *P* = 0.0743; Fig. 6J). Additionally, sex differences were found for period (Sex, *F*_(1, 4)_ = 25.88, *P* = 0.0070) and Max Qp values (Sex, *F*_(1, 4)_ = 12.64, *P* = 0.0237), but no statistically significant differences were observed for circadian disruption measures between male and female cryptochrome-deficient mice (Supp. Fig. 4).

## Discussion

Here we show the feasibility of conducting a comprehensive circadian screen within standard IVCs using the DVC system in combination with black cages with in-built cage lighting. Using well-established circadian parameters commonly employed in circadian phenotyping studies, we observed the repertoire of circadian phenotypes typically measured with standard monitoring techniques. Our results showed that mice housed within the DVC system exhibited a circadian period close to 24 h when subjected to a 12:12 LD cycle. Mice further demonstrated a free-running period of about 23.5 h under DD, and a period lengthening under LL, indicative of a robust circadian clock under DD and weaker rhythms under LL. Consistent with previous studies, mice effectively responded to phase shifts induced by interventions such as jet-lag or light pulses. Animals took 6 days to re-entrain to a 6-hour phase advance of the LD cycle, which indicates a rate of 1 day per hour shifted. Following a 2-hour LP at the beginning of the subjective night (ZT 14–16), mice delayed their circadian phase by approximately 1 h under subsequent DD.

Sex differences in period, amplitude and the stability of rhythms, as well as phase shifting responses, were also observed. Female reproductive cycles can change both activity levels and phase, with an increased activity in proestrus/estrus, which can lead to a distinctive “scalloping” pattern of activity across the days of the oestrous cycle in hamsters. However, results on these oestrous effects are more varied in mice, potentially reflecting strain, diet, housing conditions and methods of activity monitoring. Here we show subtle sex-dependent differences in home cage activity, with higher activity in females consistent with previous studies^67^. However, as our study primarily focused on responses to changing light conditions, we did not maintain mice under consistent lighting for extended periods nor perform oestrous cytology. Future studies are thus needed to relate patterns of home cage locomotor activity to the oestrous cycle using the DVC system.

Additionally, the DVC system successfully detected arrhythmic mutants and their expected phenotypic differences. Our results showed that under LD conditions, Cryptochrome-deficient mice exhibited no differences in period compared to wildtype controls, yet they displayed increased activity during the light phase and earlier activity onsets. Under DD, cryptochrome-deficient animals were arrhythmic, as expected for mice lacking a functional circadian clock. An important issue when adopting a different approach to circadian activity monitoring is its performance against other methods, such as running wheels, particularly when comparing its ability to detect differences between experimental groups. For mutagenesis screens, published data from C57BL/6J mice *tau* are 23.69 h ± 0.06^73^ and 23.7 h ± 0.17^74^, which is comparable to that observed here in the DVC (23.50 h ± 0.07). This suggests that it should be possible to reliably detect phenodeviants in forward genetic screens (typically defined as 3 standard deviations from the mean). Where comparisons are made between two groups (e.g. wildtype vs. knockout), power calculations based on α = 0.05 and power (1-β) = 0.80, show that detecting an effect size (Cohen’s d) of 2 requires 6 animals per group. For the DVC data, this would translate to detecting a 0.13 h difference in *tau* between groups with *N* = 6 per group. This is again comparable to running wheels and sufficient to detect most transgenic lines with all but the most subtle circadian phenotypes.

Collectively, these findings show that the DVC system provides a versatile tool for quantifying circadian disruption, investigating clock gene mutants, and facilitating comprehensive circadian phenotyping. Moreover, this is, to our knowledge, the first example of a comprehensive circadian screen conducted using IVCs.

As with any home cage activity monitoring system, the DVC possesses both strengths and limitations that potential users should consider (Table 1). These are detailed below.

**Table 1:** Strengths and limitations of the DVC and Leddy systems for circadian rhythm studies.

Strengths	Limitations
High-capacity monitoring	Daily observation logistics
Individual light-dark cycle control	Leddy battery power
Enhanced biosecurity	Leddy configuration
Environmental enrichment compatibility	Absence of light sensor
Environmental monitoring	Additional analytics
Wheel-running integration	Cost
Real-time data accessibility	Research flexibility

### Strengths

1. *High-capacity monitoring*. An advantage of the DVC system is its high capacity, allowing for the simultaneous monitoring of up to 80 individual cages. Circadian rhythm studies often utilise light-tight chambers or “coffins”, which typically house between 6 and 10 animals per chamber. The DVC system offers a space-efficient alternative, as one 80 cage DVC rack occupies the space equivalent of a stack of four typical chambers, which would hold between 24 and 40 cages (2–3 times stocking density). By maximizing laboratory space utilisation, the DVC system is an advantageous solution for institutions with limited animal facility space.
2. *Individual LD cycle control*. Each cage in the DVC system can be programmed to operate under its own light-dark cycle. The Leddy lighting system allows for adjustable light intensity, duration, dawn-dusk transitions and non-24 h LD cycles, simulating a wide range of lighting conditions. This provides researchers with a high level of experimental flexibility and customisation.
3. *Enhanced biosecurity*. The use of IVCs can also help prevent the spread of animal pathogens and reduce staff exposure to laboratory animal allergens, as opposed to open-top cages. These are key factors in the widespread adoption of IVCs in laboratory animal husbandry.
4. *Environmental enrichment compatibility*. The incorporation of environmental enrichment, such as nesting materials and tubes, does not limit the detection of animal movement by the DVC system, unlike other methods such as PIR recordings and video tracking where the animal must be in view throughout.
5. *Environmental monitoring*. The DVC system can also provide alerts for cage disturbances such as low food or water, water bottle leaks and soiled bedding conditions, as well as deviations from normal activity patterns. This ensures the reliability and accuracy of experimental data by promptly identifying potential disruptions as well as providing a useful welfare check for researcher and animal care staff. The DVC also timestamps any cage removal and replacement in the rack. For example, this can be used to correlate daily welfare checks and other non-photic cues with small sudden changes in behaviour. Additionally, the Rack Environmental Monitoring (REM^®^) device can monitor environmental parameters outside the rack such as temperature, humidity, noise, light, vibration and human presence. This also enables users to receive alerts and correlate these parameters to activity metrics to determine how facility disturbances may affect data. This environmental monitoring may benefit the reproducibility of circadian and behavioural studies by enabling detection of changes in the environment that may influence animal behaviour.
6. *Wheel-running integration*. Running wheels can be integrated into the DVCs, providing additional opportunities for the monitoring of voluntary activity and exercise behaviour in a manner comparable with the majority of published circadian studies.
7. *Real-time data accessibility*. The DVC system provides researchers with access to real-time data through its cloud-based DVC Analytics platform. This feature allows for continuous monitoring of animal behaviour from any location, facilitating timely intervention, data analysis and storage. The platform offers a range of parameters for data inspection and visualization, including single actograms. The DVC system measures home cage locomotor activity, distance, and velocity, as well as variables relevant to circadian rhythm studies, such as environmental disturbances and alerts for potential behavioural impacts. This comprehensive data collection may enable researchers to gain insights into the complex interplay between circadian rhythms and laboratory environmental factors. Critically, real-time analytics enable researchers and technicians to respond to changes in activity patterns, for example if animals are sick.

Despite its strengths, the current format of the DVC system does have several potential limitations that circadian researchers should be aware of.

### Limitations

1. *Daily observation logistics*. The benefits of using black cages as independent light-tight chambers must be weighed against practical considerations of their use. Black cages require specific protocols for daily animal checks, similar to light-tight chambers, where cages must be opened at appropriate phases of the LD cycle. When room lights match the cages’ light cycles, checks can be performed during the light phase. Similarly, if cages are under constant darkness, they can be observed when the room is in darkness using dim red light or infrared goggles. If the room lights and the cages’ light cycles cannot be aligned, cages must be removed from the rack and observed in a different room to avoid light exposure. Where complex lighting schedules are required or multiple users require different lighting schedules, this may be a logistical challenge to manage. Researchers should be aware of these limitations when considering using the DVC system.
2. *Leddy battery power*. One drawback of the Leddy lighting system is its requirement to be removed from the cage for charging. The air-tight sealed cages prevent the use of cables and the system cannot operate while charging. This necessitates additional Leddy systems to be switched in to maintain experimental conditions. Under a 12:12 LD cycle at ~ 100 lx, a Leddy battery can last up to three weeks. However, the non-linear battery life necessitates regular checks and maintenance, which may pose an additional logistical challenge for researchers.
3. *Leddy configuration*. The Leddy lighting system is unable to accommodate complex LD cycles. Its configuration allows for changes between light and dark phases, including dim light ramps, but not consecutive periods of light at different intensities, such as dim evening light^75^. Moreover, the system only provides cool white LEDs, precluding any adjustments to the light spectrum. Incorporating additional lighting components, such as red LEDs for welfare checks or violet LEDs to help simulate daylight, would further enhance its versatility and functionality^61^.
4. *Absence of light sensor*. Individual light sensors are not present within each cage, necessitating careful monitoring to ensure data accuracy and consistency of lighting schedules.
5. *Additional analytics*. The inclusion of double plotted actograms and standard measures of circadian disruptions within the DVC Analytics platform would enhance its utility for circadian researchers. This would allow comprehensive data analysis and interpretation on a single platform. For example, periodogram analysis and activity onset detection, as well as key metrics of circadian disruption such as IS, IV and RA would be particularly beneficial.
6. *Cost*. The initial investment needed to deploy the DVC and Leddy lighting systems represents a substantial capital investment, with additional expenses incurred for ongoing analytics. Consequently, this approach may be better suited for core research facilities that accommodate multiple research groups rather than individual labs. Increasing interest from labs working on mouse behaviour may also facilitate such integration within core facilities. Whilst costs are decreasing with increasing uptake, users should be aware of overheads associated with this technology.
7. *Research flexibility*. The standard IVC cage format may not be suitable for researchers requiring greater flexibility in their experimental design. For example, researchers using non-standard caging, complex lighting systems, cameras/sensors, telemetry, tethered recordings, or in-cage behavioural measurements may preclude the use of an IVC.

As can be seen from the above, the DVC system offers many advantages, including high-capacity monitoring, individual LD cycle control, enhanced biosecurity, environmental monitoring features, high resolution, real-time accessibility and user-friendly online analytics. However, new users should consider potential limitations, such as the absence of light sensors and the charging logistics of the Leddy system, that may affect its performance in circadian rhythm studies. Additionally, research flexibility may be key. The DVC system might not work for researchers aiming to perform complex light cycle manipulations or specific types of studies where an IVC format is unsuitable.

Here we show that the DVC system provides an alternative tool for circadian phenotyping, providing an opportunity to conduct such assessments in IVC cages. The storage and real-time availability of the data on the cloud-based DVC analytics platform facilitates data analysis, as well as ongoing experimental monitoring and interventions, providing an advantage over existing systems. However, researchers should be aware of the current limitations particularly with regard to cost and research flexibility, which may influence the adoption of an IVC based system. In this regard, the DVC system may be more suited for central phenotyping facilities rather than individual circadian labs.

## Electronic supplementary material

Below is the link to the electronic supplementary material.

Supplementary Material 1

## Data Availability

Raw activity data with metadata for all data are available on GitHub (https://github.com/selmatir/DVC-Circadian-Phenotyping).
