# Supplementary material for: Evaluation of the Digital Ventilated Cage® system for circadian phenotyping
Source: Sci Rep. 2025 Jan 29;15:3674. doi: 10.1038/s41598-025-87530-6 (PMC11779816; doi:10.1038/s41598-025-87530-6)
Supplement: Supplementary file 1 — Supplementary Material 1 [file 41598_2025_87530_MOESM1_ESM.docx]

**Supplemental Information**


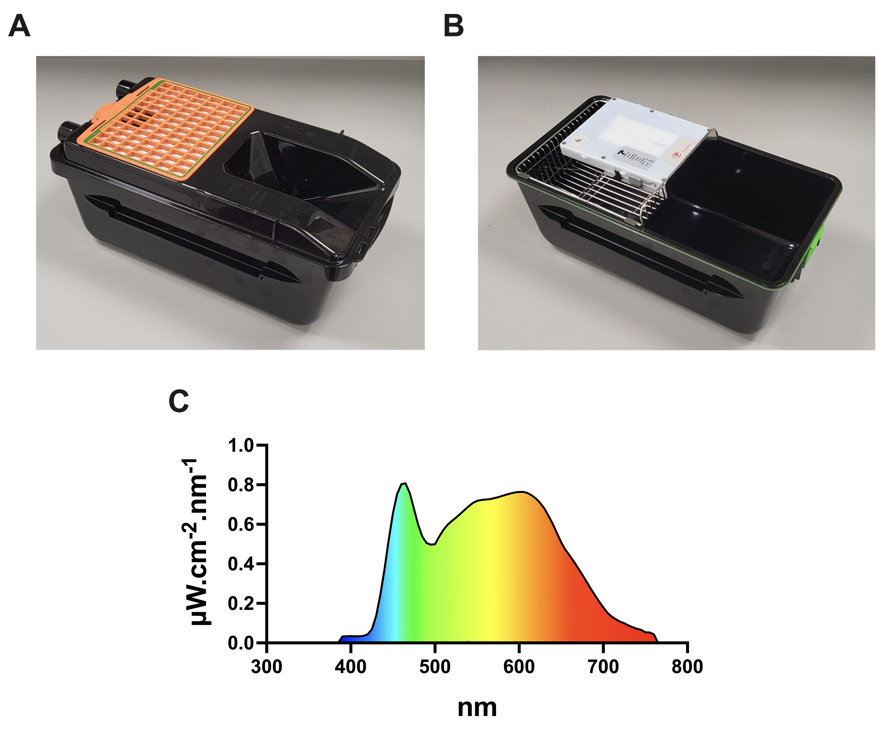


**Supplemental Figure 1.** Black DVC cage and Leddy lighting system. A. Closed view and B. Open view of a black DVC cage equipped with a Leddy lighting system. C. Spectral power distribution chart of the Leddy at light intensity level 22.

**Supplemental Table 1.** Spectral power distribution of the Leddy lighting system at light intensity level 22.

| nm | µW.cm^-2^.s^-1^ | nm | µW.cm^-2^.s^-1^ |
| --- | --- | --- | --- |
| 300 | 0 | 545 | 0.707907428 |
| 305 | 0 | 550 | 0.718842202 |
| 310 | 0 | 555 | 0.722735247 |
| 315 | 0 | 560 | 0.725142483 |
| 320 | 0 | 565 | 0.727482315 |
| 325 | 0 | 570 | 0.73192321 |
| 330 | 0 | 575 | 0.737395487 |
| 335 | 0 | 580 | 0.743373588 |
| 340 | 0 | 585 | 0.750512991 |
| 345 | 0 | 590 | 0.756208319 |
| 350 | 0 | 595 | 0.761354284 |
| 355 | 0 | 600 | 0.763915072 |
| 360 | 0 | 605 | 0.763767282 |
| 365 | 0 | 610 | 0.757710251 |
| 370 | 0 | 615 | 0.746227219 |
| 375 | 0 | 620 | 0.729391235 |
| 380 | 0 | 625 | 0.707889267 |
| 385 | 0 | 630 | 0.679822289 |
| 390 | 0.032529912 | 635 | 0.642123981 |
| 395 | 0.033849468 | 640 | 0.601247593 |
| 400 | 0.034693374 | 645 | 0.554548111 |
| 405 | 0.033590906 | 650 | 0.508052821 |
| 410 | 0.033575219 | 655 | 0.471222214 |
| 415 | 0.035761695 | 660 | 0.442100136 |
| 420 | 0.043843236 | 665 | 0.412491179 |
| 425 | 0.069125606 | 670 | 0.379144738 |
| 430 | 0.136052666 | 675 | 0.345627195 |
| 435 | 0.241369271 | 680 | 0.310587784 |
| 440 | 0.373673509 | 685 | 0.275717379 |
| 445 | 0.520506524 | 690 | 0.241339905 |
| 450 | 0.656460295 | 695 | 0.209853461 |
| 455 | 0.752857712 | 700 | 0.179711496 |
| 460 | 0.80319366 | 705 | 0.152475128 |
| 465 | 0.807369011 | 710 | 0.133126363 |
| 470 | 0.757781439 | 715 | 0.11938742 |
| 475 | 0.6797518 | 720 | 0.106866188 |
| 480 | 0.596800237 | 725 | 0.099167912 |
| 485 | 0.537800661 | 730 | 0.08790518 |
| 490 | 0.505381555 | 735 | 0.079725687 |
| 495 | 0.497358909 | 740 | 0.07128311 |
| 500 | 0.499728078 | 745 | 0.06588531 |
| 505 | 0.538099965 | 750 | 0.053602231 |
| 510 | 0.572088873 | 755 | 0.052955424 |
| 515 | 0.598159677 | 760 | 0.043976372 |
| 520 | 0.617308833 | 765 | 0 |
| 525 | 0.633836375 | 770 | 0 |
| 530 | 0.652874762 | 775 | 0 |
| 535 | 0.672169495 | 780 | 0 |
| 540 | 0.691301073 |  |  |


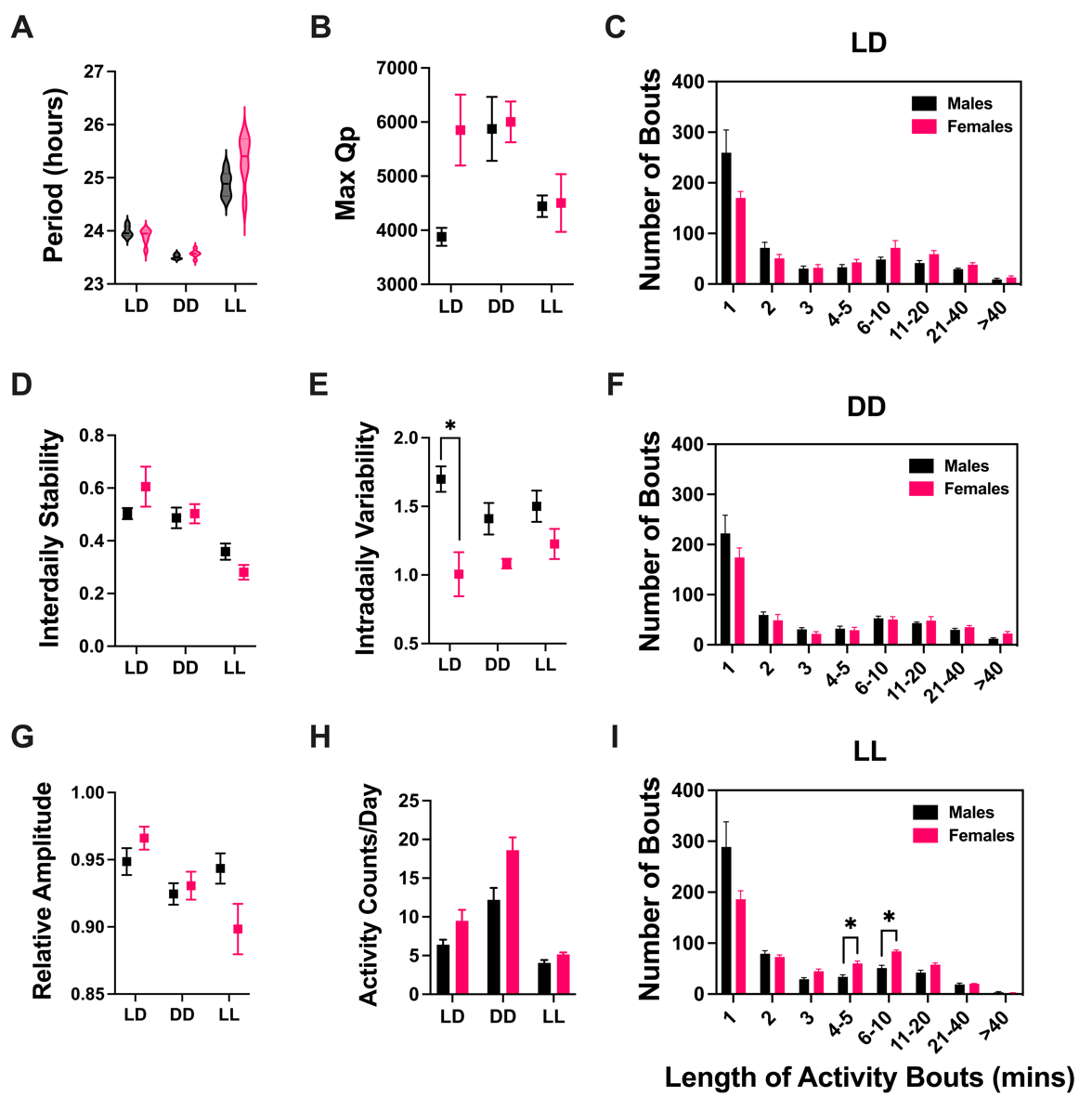


**Supplemental Figure 2**. Disaggregation of circadian disruption measures by sex for 6 male and 6 female wildtype C57BL/6J mice under 12:12 Light-Dark (LD), constant darkness (DD) and constant light (LL). A) Period of activity rhythms (Sex, *F*_(1, 10)_ = 4.313, *P* = 0.0645). B) Maximum Qp values (Sex, *F*_(1, 10)_ = 4.033, *P* = 0.0724). C) Distribution of the number and duration of activity bouts in LD (Bout length x Sex, *F*_(7, 70)_ = 4.091, *P* = 0.0008). D) Inter-daily Stability (Sex, *F*_(1, 10)_ = 0.1131, *P* = 0.7436). E) Intra-daily Variability (Sex, *F*_(1, 10)_ = 14.52, *P* = 0.0034). F) Distribution of the number and duration of activity bouts in DD (Bout length x Sex, *F*_(7, 70)_ = 1.451, *P* = 0.1991). G) Relative Amplitude (Sex, *F*_(1, 10)_ = 0.4046, *P* = 0.5390). H) Total activity per day (Sex, *F*_(1, 10)_ = 14.85, *P* = 0.0032). I) Distribution of the number and duration of activity bouts in LL (Bout length x Sex, *F*_(7, 70)_ = 5.040, *P* = 0.0001). Mean +/- SEM. Statistically significant multiple comparisons are indicated by an asterisk.


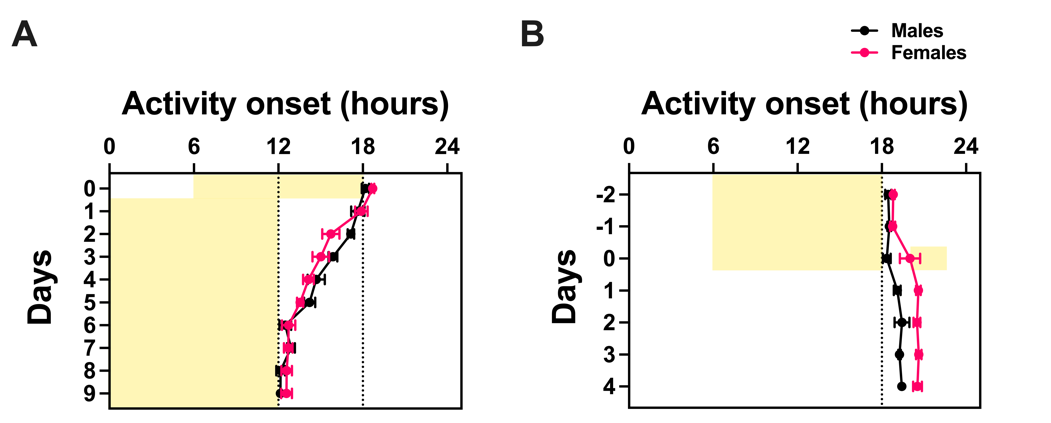


**Supplemental Figure 3**. Disaggregation of phase shifting responses by sex for 6 male and 6 female wildtype C57BL/6J mice. A) Both sexes required about 6 days to re-entrain to the Light-Dark (LD) cycle following a 6-hour phase advance, as illustrated by the shift in activity onset (Sex, *F*_(1, 10)_ = 0.5986, *P* = 0.4570). B) Females exhibited a greater delay in activity onset by about 2 fold in constant darkness (DD) following exposure to a light pulse at ZT14-16, as compared to males (Sex, *F*_(1, 10)_ = 15.47, *P* = 0.0028). Mean +/- SEM.


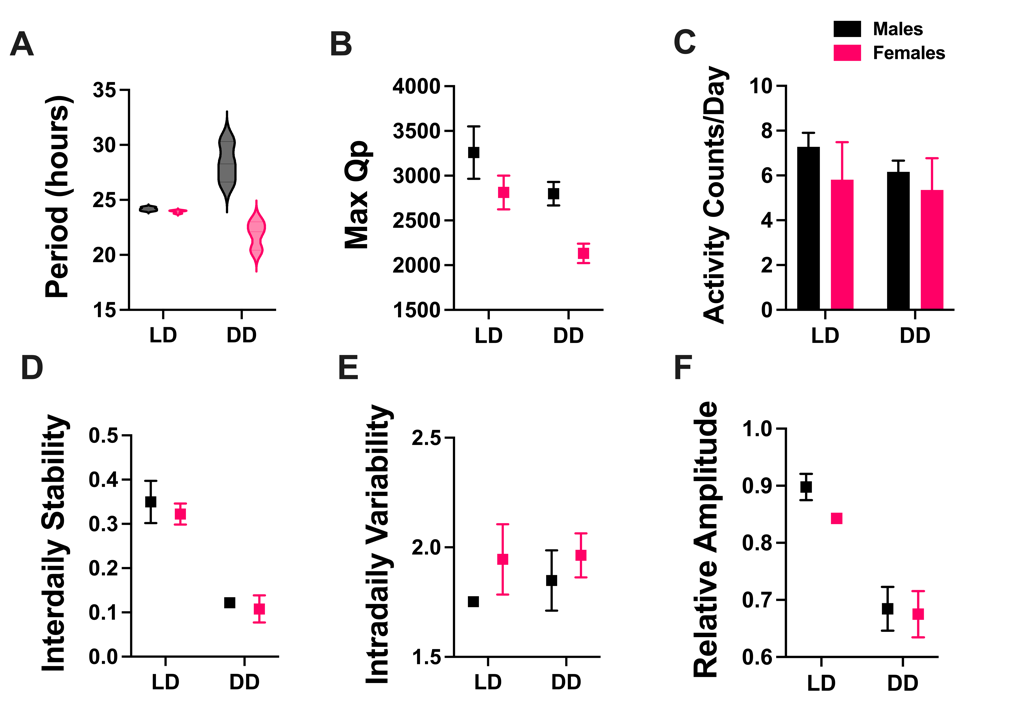


**Supplemental Figure 4**. Disaggregation of circadian disruption measures by sex for 3 male and 3 female Cryptochrome-deficient mice under 12:12 Light-Dark (LD) and constant darkness (DD). A) Period of activity rhythms. B) Maximum Qp values (Sex, *F*_(1, 4)_ = 12.64, *P* = 0.0237). C) Total activity per day (Sex, *F*_(1, 4)_ = 0.5731, *P* = 0.4912). D) Inter-daily Stability (Sex, *F*_(1, 4)_ = 0.3424, *P* = 0.5899). E) Intra-daily Variability (Sex, *F*_(1, 4)_ = 1.174, *P* = 0.3396). F) Relative Amplitude (Sex, *F*_(1, 4)_ = 1.445, *P* = 0.2956). Mean +/- SEM.
